# Timing for antioxidant-priming against rice seed ageing: optimal only in non-resistant stage

**DOI:** 10.1038/s41598-020-70189-6

**Published:** 2020-08-06

**Authors:** Ling-xiang Xu, Xia Xin, Guang-kun Yin, Ji Zhou, Yuan-chang Zhou, Xin-xiong Lu

**Affiliations:** 1 National Crop Genebank, Institute of Crop Science, Chinese Academy of Agricultural Sciences, Beijing, 100081 People’s Republic of China; 2 Key Laboratory of Ministry of Education for Genetics, Breeding and Multiple Utilization of Crops, College of Crop Science, Fujian Agriculture and Forestry University, Jinshan, 350002 Fuzhou People’s Republic of China; 3 Plant Phenomics Research Center, Nanjing Agricultural University, Nanjing, 210095 People’s Republic of China

**Keywords:** Plant physiology, Heat

## Abstract

Seed deterioration due to ageing strongly affects both germplasm preservation and agricultural production. Decelerating seed deterioration and boosting seed viability become increasingly urgent. The loss of seed viability is inevitable even under cold storage. For species with short-lived seed or for regions with poor preservation infrastructure where cold storage is not readily available, seed enhancement is more reliable to increase seed viability and longevity. Antioxidant priming as a way of seed enhancement usually improves seed germination. As for post-priming survival, however, significant uncertainty exists. The controversy lies particularly on seeds of high germination percentage (GP > 95%) whose viability is hardly improvable and the benefits of priming depend on prolonging seed longevity. Therefore, this study timed antioxidant priming to prolong the longevity of high-viability seeds under artificially accelerated ageing (AAA). Rice (Nipponbare) seeds (GP > 97%) under room-temperature-storage (RTS) for 6 months. were resistant to AAA first with little viability loss for a certain period, the resistant stage. This resistance gradually vanished without GP change, during a prolonged RTS period which was named the vulnerable stage. According to the results, although antioxidant priming severely curtailed the resistant stage for seeds with a long plateau in the survival curve, it decelerated viability loss for seeds in the vulnerable stage. In complement to seed storage, priming potentially retains high seed GP which would decrease without seed enhancement. To maximize the benefits of priming for high-GP seeds, two time points are advised as the start of a time window for priming: (1) just at the end of the resistant stage without notable viability loss, which is hard to grasp by GP monitoring; (2) slight but identifiable GP decline.

## Introduction

Inevitable deterioration with the passing of time is a limitation for seed preservation. Seed deterioration threatens both germplasm conservation and agricultural production^1,2^. Boosting both seed longevity and germination is therefore of pressing significance for seed preservation^2^. In ex situ germplasm storage, seed is stored at low temperatures, generally around − 18 °C but also close to freezing (0–4 °C), and low seed moisture, usually in equilibrium with 15–25% relative humidity^3^. Under cold storage, seed longevities above the threshold (~ 85%) are usually more than decades^3^.

Seed ageing can be roughly divided into two phases as time passes: (1) the resistant stage during which seeds have high and more or less stable germination; and (2) the vulnerable stage during which seeds rapidly lose viability and ageing-resistance. This trend applies to seeds whose original ageing-resistance is high during cold storage^4^, air temperature storage^5^, or under artificial accelerated ageing (AAA) experiments^6^ where a “plateau” at the early stage of a survival curve is identifiable. Therefore, prolonging the resistant stage and decelerating viability loss at the later stage are the main purposes for improving seed storage.

It is more challenging for a seed to be stored under some sub-optimal or even harsh environment where cold storage is unavailable and the resistant stage cannot be long. In warm or humid areas, especially among less developed countries with less advanced infrastructure, the decline of seed viability is more sever^7–9^. Even worse, various wild species seeds are short-lived^10,11^. When longevity under storage is short, or when seeds inevitably begin to deteriorate rapidly, seed enhancement^12^ which has the potential to retain seed viability may serve as a complement to storage. One popular form of seed enhancement is priming which contains a hydrate-dehydrate process before sowing to invigorate seeds and promote germination^13^, hence its name “priming” which means promotion at the start.

Seed deterioration as a result of ageing is attributed to the imbalance in the reductive /oxidative (redox) state caused by the accumulation of reactive oxygen species (ROS) which accelerate viability loss^8,14–16^. Antioxidant priming has been shown to be effective in removing ROS^17^, boosting antioxidant enzymes^17^, and, through inhibition of lipid oxidation, increasing cell membrane integrity (CMI) as measured by electrical conductivity (EC)^18^. The antioxidant effect is achieved by holding the seed in a hydrated state before radicle protrusion^19^. This state is to make full use of antioxidant enzymes and antioxidants to scavenge ROS^6^ and to apply exogenous antioxidants during priming^20^.

During storage the seed is dehydrated and antioxidant enzymes are inactive^21^. Antioxidant priming is therefore beneficial to the activation of antioxidant system. However, its exact role in subsequent storage, indicated by post-priming seed survival, is much more ambiguous^6^. One probable reason is that it promotes radicle-protrusion during which a seed loses its desiccation tolerance^22^ and therefore suffers cell membrane injury during desiccation^21^. In practice there is hardly any standard for priming as a method to prolong seed longevity^20^.

Could antioxidant priming act as a regular method of boosting seed longevity, and if so, when should a seed lot be primed? Priming has been found to be more likely to benefit seeds at the vulnerable stage^6^. Our question was whether would antioxidant priming prolong or abbreviate the resistant stage? Would it decelerate viability loss? To better understand the exact role of antioxidant priming, antioxidant^15^ and oxidant priming were applied to rice seeds to study post-priming survival. Rice is the model species for cereals and ranks third in the world’s crop production, following maize and wheat. It is also the staple food in tropical or subtropical areas where temperatures are high. Without cold storage, environments for seed preservation are inclement and seed enhancement can be more useful. The original germination percentage (GP) of rice (Nipponbare, NPB) seeds in this study was already 97%, hard to improve. So, priming could only benefit seeds through prolonging the resistant stage or decelerating viability loss rather than increasing GP. Two hypotheses were proposed: (1) ageing resistance significantly declines as a result of storage before GP decreases; (2) antioxidant priming increases ageing resistance at high GP and decelerates viability loss. This study sought to discover an optimal time point for seed priming against viability loss, which can be generalized to other species and circumstances of storage and perhaps extended to be incorporated with other types of seed enhancement.

## Results

### Seed longevity under AAA responded negatively to the duration of RTS: the loss of artificial-ageing resistance was before identifiable change of germination

**Figure 1:**
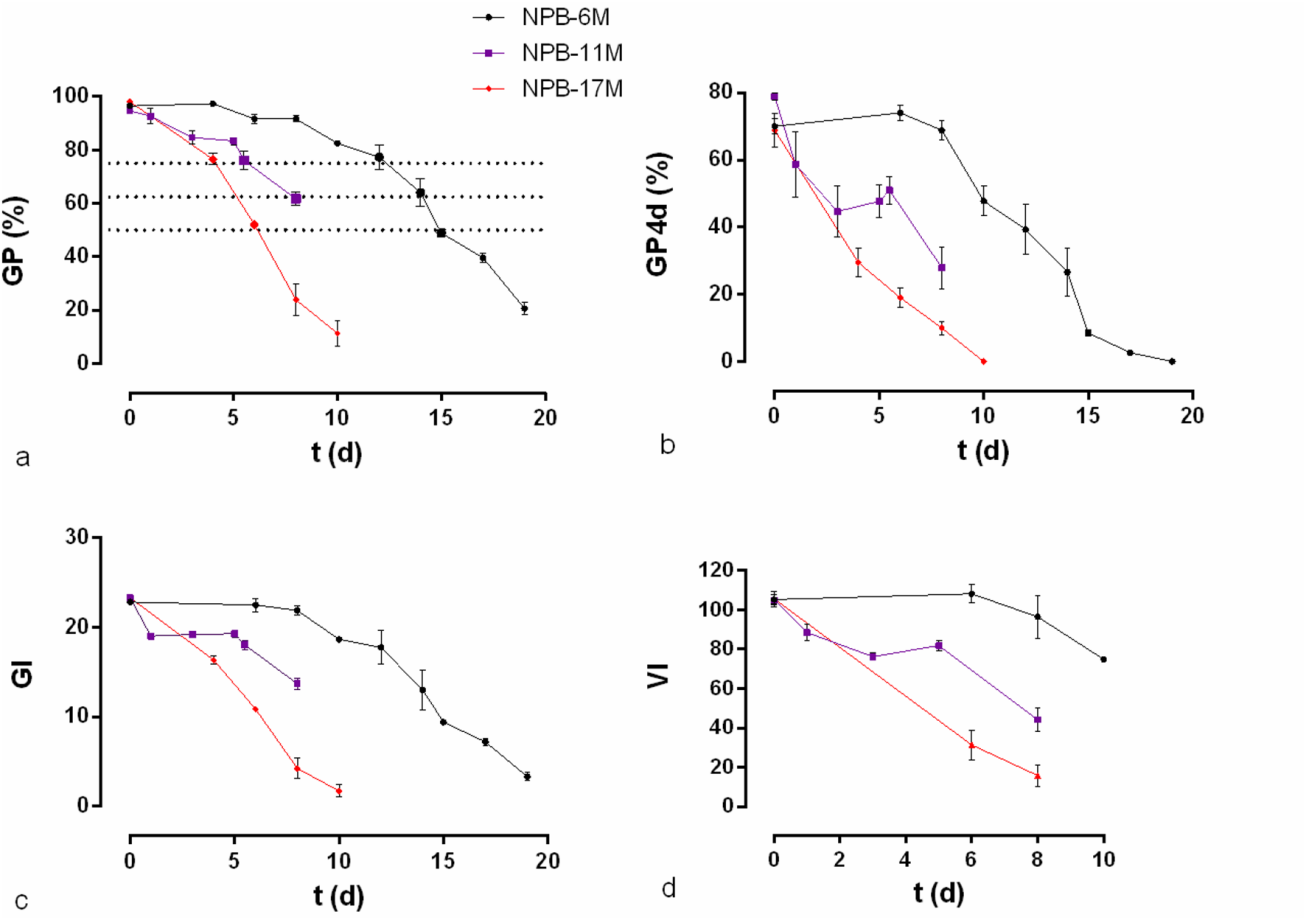
Effects of the duration of room-termperature storage on the seed survival curves under artificial accelerated ageing. NPB-6M, -11M and -17M represented the rice (Nipponbare) seeds stored under room temperature for 6 months, 11 months, and 17 months, respectively. Deterioration curves of germination percentage (GP, **a**), germination power (GP4d, **b**), germination index (GI, **c**) and vigor index (VI, **d**) were for the 3 NPB samples. Dashlines (between larger plots) highlighted the timespan difference to deteriorate to similar viabilities, marking GP = 75%, 62.5% and 50%. Standard error was labeled by bar.

NPB-6M (6-months RTS, Table 1) experienced the “plateau” stage with a slight fluctuation (Fig. 1a) at the early stage of AAA for ~ 8 days. To the quarter-mortality level (GP > 75%) NPB-6M had already the significantly longer longevity due to its reasonably long “plateau” in its survival curve and at this point of quarter-mortality, all 3 samples loss viability significantly more rapidly. NPB-6M’s GP was maximum at 4 days and still above 90% at 8 days. In contrast, NPB-17M (17-months RTS, Table 1) dropped to ~ 30% at 8 days despite the same initial GP (Fig. 1a). NPB-11M (11-months RTS, Table 1) deteriorated significantly faster than NPB-6M and slower than NPB-17M. This general trend in GP across the samples was reflected in other germination/vigor indices as GP4d (GP at 4 days since sowing), GI (germination index) and VI (vigor index) (Fig. 1). NPB-6M’s GP4d and VI also increased with ageing and was maximum at 6 days (Fig. 1d). NPB-11M had the highest GP4d among all samples at 0 days and, except for 0 days, GI declined at 1 day but then did not decline further until after 6 days (Fig. 1c). RTS influenced the longevity in comparison with the GP from 6 to 17 months of storage. NPB-6M was resistant to AAA because by fitting the survival curve of NPB-6M to that of − 11M and − 17M, NPB-6M’s GP declined 6 days (R^2^ = 0.9658) and 8 days (R^2^ = 0.9586) later respectively under AAA (Fig. S1).

**Table 1:** Description of treatments and usage of samples in addition to drawing survival curves.

	Start of RTS	RTS until– (duration)	Usage of seed materials	
NPB-6M	Nov. 2015	May 2016 (6 months)	NPB-6M-S1/HP/H100, no post-priming AAA	Analyzing intial ROS level, cell membrane integrity before AAA
			NPB-6M-S1/HP/H100-6d Post- priming AAA	ROS level after AAA
NPB-17M	Nov. 2015	Apr. 2017 (17 months)	Determination of solutes and duration of AAA for NPB-6M	
NPB-11M	Oct. 2016	Sep. 2017 (11 months)	Determination of the most sensitive tissue to ageing for cell membrane integrity test in NPB-6M	

Abbreviation of treatment: HP, S1, H100: priming for ~ 24 h with distilled water, 1 mM spermidine, 100 mM hydrogen peroxide respectively. Accelerated artificial ageing (AAA) at 40 °C and 75% relative humidity (RH) were exerted for various durations, e.g.: 6 days on NPB-6M-6d or NPB-17M-6d, 15 for NPB-6M-15d, and so on for – 4 days, − 8 days, − 17 days and other samples. NPB-17M-S1-6d means NPB-17M experienced priming for ~ 24 h with 1 mM spermidine and then 6 days AAA. Post-priming germination tests were performed on NPB-6M and NPB-17M. Membrane integrity and ROS tests were exerted on NPB-6M samples including primed samples and post-priming AAA samples to check the factors affecting seed storage. Before the start of RTS seeds were deposited at − 18 °C.

### 1-mM-spermidine solution proved to be beneficial for post-priming survival in NPB-17M

According to the data in Tab. S1, 0.25 mM and 5 mM spermidine (spd) were excluded from the suitable concentration for priming, as they already showed a detrimental effect on GP even without subsequent AAA. Therefore, post-priming AAA treatments only included 0.5, 1, 2, and 3 mM. Solutions were further selected according to the GP and VI of the seed samples after 10-days AAA. Finally, 1 mM spd significantly surpassed other concentrations after 6-days AAA (Table 2), hence its anti-ageing use in later tests.

**Table 2:** Effects of 6-days post-priming artificial ageing on seeds.

	N	WC ± SE (%)	N	GP ± SE (%)	N	VI ± SE
NPB-17M-H100-6d	3	8.873 ± 0.401	**4**	**41.00 ± 2.22 c(c)**^**#**^	**4**	**28.07 ± 2.25 d(d)**
NPB-17M-H50-6d	3	n	2	38.00 ± 2.00 c(c)^#^	2	26.06 ± 5.84 d(d)
NPB-17M-S3-6d	3	8.213 ± 0.036	2	47.00 ± 7.00 c(c)	2	33.86 ± 5.02 c(cd)
NPB-17M-S2-6d	3	8.524 ± 0.109	3	63.33 ± 2.40 b(ab)*	3	47.21 ± 5.20 bc(bc)
NPB-17M-S1-6d	4	8.253 ± 0.453	**3**	73.33 ± 4.67 a(a)**	**3**	**70.03 ± 1.29 a(a)**
NPB-17M-S0.5-6d	3	8.100 ± 0.212	2	48.00 ± 2.00 c(bc)	2	35.36 ± 2.30 c(cd)
NPB-17M-HP-6d	2	8.256 ± 0.178	3	64.00 ± 3.06 b(ab)*	3	56.07 ± 2.21 b(b)
NPB-17M-6d			2	52.00 ± 0.00 f.h	2	41.42 ± 2.97c(bcd)

*n.s.* not significant, *f.h.* data which caused failure of homogeneity test in variance analysis was excluded from multiple comparison test, *AAA* artificial accelerated ageing, *NP* no priming, *HP* hydropriming, *GP* germination percentage, *SE* standard error, *VI* vigor index, *H100, 50* priming with 100, 50 mM hydrogen peroxide, *S5–S0.25* priming with 5 mM to 0.25 mM spermidine. NPB-17M-S1-6d and NPB-17M-H100-6d were for further studies such as physiological and morphological studies, and there *GP* and *VI* were labelled in bold.

Different letters indicated that the levels were significantly different with P < 0.05 (extremely significantly with P < 0.01 for letters within the brackets).

*,**: significantly higher than non-primed control at P < 0.05, 0.01 level respectively.

#: significantly lower than control at P < 0.05. GP was arcsin-transformed in multiple comparison test, but its original value kept still in this table.

The duration of 6 days was deduced according to Fig. S2 where only at 6 days was the GP midst 75% and 40%. When the GP was near 40% many treatments showed no significant difference to others at 10 days (Tab. S1), and 8 days was not selected because NPB-17M-HP-8d’s GP came close to many treatments at 10 days of ageing, including NPB-17M-HP-10d. Spd pretreated NPB-17M might lose GP from 8 to 10 days as slowly as NPB-17M-HP-8d, hence their difference which may not be significant. Above 75%, seeds were unlikely to deteriorate rapidly and their GP came somewhat close to each other. For H_2_O_2_, 50 mM and 100 mM as oxidative treatments were experimentally recommended by our colleagues in other studies where these concentrations did exert oxidative stress and 100 mM was selected in this study (Tab. S2).

**Figure 2:**
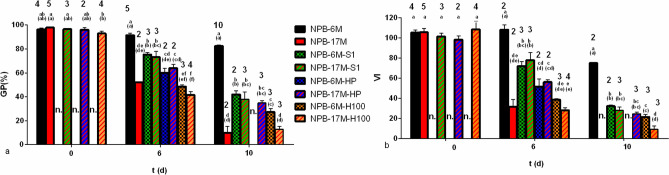
Effect of pretreatments upon seed survival under artificial accelerated ageing at 0 day, 6 days and 10 days. NPB-6M and -17M represented the rice (Nipponbare) seeds stored under room temperature for 6 months and 17 months, respectively. *S1* priming with 1 mM spermidine; *HP* hydropriming; *H100* priming with 100 mM H_2_O_2_. Different letters indicated that the levels were significantly different with P < 0.05 (extremely significantly with P < 0.01 for letters within the brackets). GP was arcsin-transformed in multiple comparison test, but its original value kept still in this figure. The size of the samples, replicate numbers were labelled above the letters. Comparisons were among samples under the same duration of AAA in **(a)** and **(b)**. Standard error was labeled by bar. n., not tested.

For NPB-6M-S1-10d and NPB-6M-H100-10d, their GP was close to that of NPB-17M-S1-10d and NPB-17M-H100-10d, respectively (Fig. S3). 1 mM spd and 100 mM H_2_O_2_ seemed effective for testing NPB-6M at 6 days, supposing that NPB-6M and -17M would deteriorate at similar speeds, if they were primed in the same solution (Fig. 2, deduced from Tab. S3, Fig. S3).

### Antioxidant priming extenuated oxidative stress but abated artificial-ageing resistance for seeds still at the resistant stage according to NPB-6M’s and -17M’s survival curves

**Figure 3:**
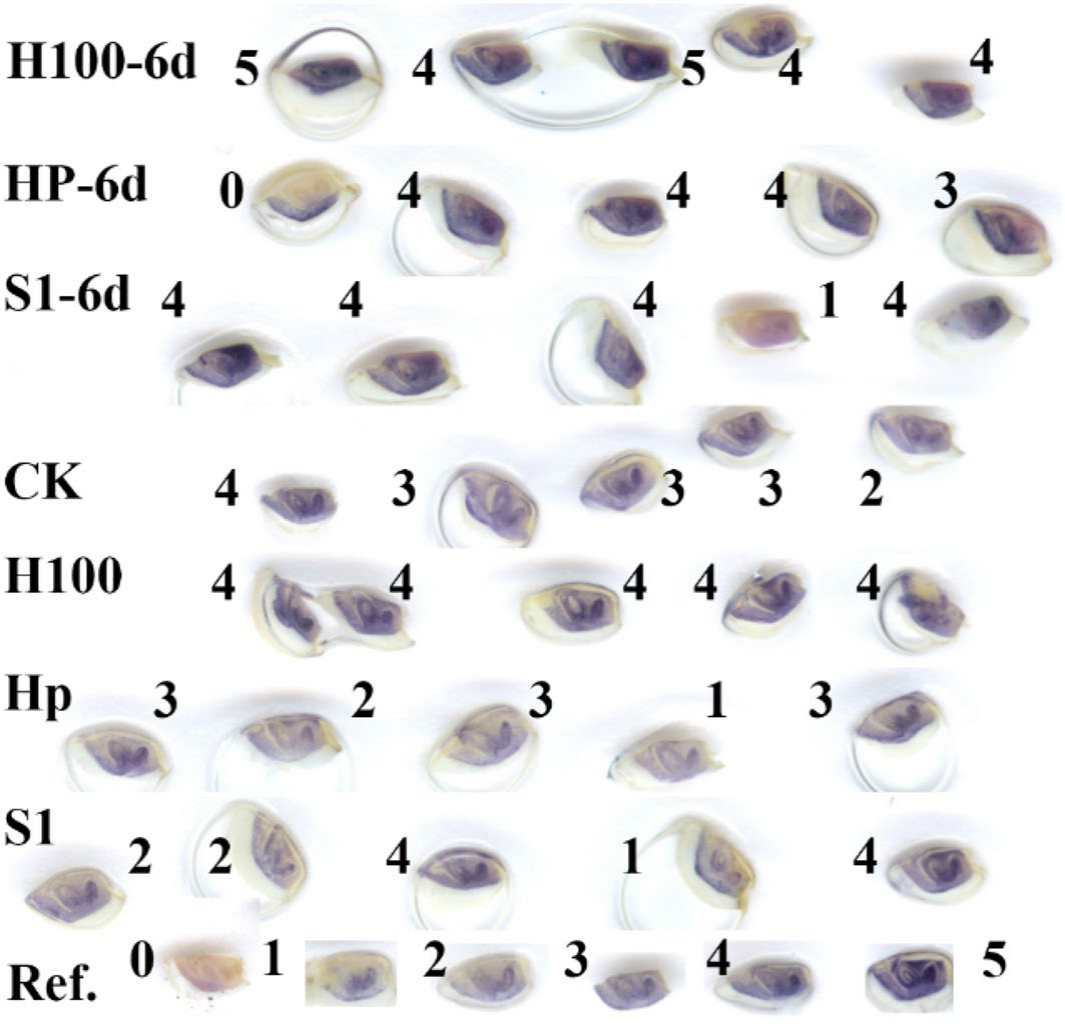
ROS level responding to priming and ageing. All samples were NPB-6M, rice (Nipponbare) seeds stored under room temperature for 6 months. H100, H100-6d, priming with 100 mM H_2_O_2_ plus 0-d or 6-d artificial ageing, and so on for HP, HP-6d, S1, S1-6d. Ref., reference for values: 0–5. 0, no apparent staining; 1, radicle and embryo axis were stained; 2, radicle and plumule were moderately stained and epiplast slightly stained; 3, radicle deep, plumule and epiplast moderate; 4, radicle very deep, plumule and epiplast deep; 5, very deep on the whole. Treatments exhibited were from one photograph by scanning. They were not well arranged because the surface of scanner was wet: water was sprayed to make the photograph clear and some embryos were embedded by droplets.

At 0 day, antioxidant priming showed no significant effect. At 6 days of AAA, every pretreatment including non-primed NPB-6M showed significant differences in GP (Fig. 2a) and VI (Fig. 2b) from each other with GP ranging from ~ 40 (H_2_O_2_-priming) to 93% (no-priming). NPB-17M-6d had similar GP and VI as NPB-H100-6d. Maximum values of GP and VI at 6 days were from non-primed NPB-6M. Redox state of priming (Fig. 3) showed significant differences between priming treatments (Fig. 2). Among all primed samples, 1 mM spd-primed NPB showed the highest GP and VI, followed by hydroprimed NPB which significantly surpassed H_2_O_2_-primed NPB. Intriguingly however, once primed in the same solution, NPB-6M and -17M failed to show significant differences either in GP or VI. It was the same at 10 days as at 6 days that antioxidant-primed seeds surpassed oxidant-primed ones, but non-primed NPB-6M retained the maximum GP (Fig. 2). Hydropriming (HP) also improved the GP of one naturally-ageing sample (from the same accession of NPB-6M and NPB-17M but was under RTS for 3 years since harvest) almost by half, from 28.29 ± 3.00 to 43.00 ± 0.50%. Further, HP also removed ROS (Fig. S4) as it did in NPB-6M-HP. However, an additional cycle of HP exhibited a detrimental effect when HP reduced NPB-6M-S1-10d’s GP from 38.00 ± 6.00 to 0%.

H_2_O_2_ exerted oxidative stress in both pre-priming and post-priming NPB-6M (Fig. 3, Table 3) in comparison to hydroprimed or spd-primed samples. HP- and spd-priming showed no significantly different effect in NPB-6M seeds both with and without AAA (Table 3). Their post-priming, pre-ageing ROS level was significantly lower than non-primed control.

**Table 3:** ROS and cell membrane permeability level of NPB-6M treatments.

ROS level				Membrane permeability	
AAA 0 day		AAA 6 days			AAA 0 day
NP	2.85 ± 0.15b			NP	0.900 ± 0.314c
S1	2.18 ± 0.15c	S1-6d	3.15 ± 0.22b	S1	1.375 ± 0.460bc
HP	2.30 ± 0.19c	HP-6d	2.92 ± 0.22b	HP	2.571 ± 0.685ab
H100	3.03 ± 0.16b	H100-6d	3.80 ± 0.17a	H100	3.000 ± 0.447a*

*NP* no priming, *NPB-6M* simply under 6 months room-temperature-storage as the control, *H100, S1, HP* priming with 100 mM hydrogen peroxide, 1 mM spermidine and distilled water respectively.

ROS level and membrane permeability level were determined by the intensity of staining by NBT and FM4-64 respectively. Different letters indicated that the levels were significantly different with P < 0.05 (extremely significantly with P < 0.01 for letters within the brackets).

*Membrane permeability of NPB-H100 was significantly greater than NP with P < 0.01.

It can be calculated that HP reduced the modelled maximum speed of losing viability (v_max_) of NPB-17M (Fig. S5). However, the reduction in oxidative stress seemed to be unnecessary to prolong AAA-simulated longevity, since non-primed NPB-6M took 6 more days (12 days vs 6 days) to decline to ~ 75% than spd-primed seeds, twice the period (Table 4) of NPB-6M-S1. The negative effect from antioxidant priming was even more severe than 5 months longer RTS since NPB-11M took 8 days to reduce to GP ≈ 60% (Table 4, Fig. 1a) while NPB-6M-HP took 6 days (Table 4).

**Table 4:** Comparison of seed deterioration rate.

	11M vs 6M		17M vs 6M		6M-S1 vs 6 M	
	GP (%)	t (d)	GP	t (d)	GP	t (d)
NPB-6M	77.33	12	77.33	12	77.33	12
NPB-11M, 17M, 6M-S1	76.00	5.5	76.50	4	75.33	6
Dif. (d)	*6.5*		*8*		*6*	
MP^75#^	**2.18**		**3.00**		2.00	
NPB-6M	60.67	14	48.86	19	39.61	17
NPB-11M, 17M, 6M-S1	61.77	8	52.00	6	41.50	10
Dif. (d)	*6*		*9*		*7*	

	11M vs 6M	17M vs 6M				
	v_max_ (% d^−1^)	v_max_ (% d^−1^)				
NPB-6M	13.13	13.13				
NPB-11M, 17M	10.61	24.76				
MP^vmax #^	**0.81**	**1.88**				

*GP* germination percentage, *Dif.* difference of t_75_ or t_~50_, highlighted in italic characters, *v*_*max*_ modelled maximum deterioration rate, *#MP*^*75*^ was the multiple of the average deterioration rate which is reciprocal of t_75_ (the timespan to deteriorate to GP≈75%), of NPB-11M, 17M and 6M-S1 respectively to reduce to 75% viability against the comparative average rate of NPB-6M, e.g. MP^75^ for “17M vs 6M” was 1/4 ÷ 1/12 = 3. Similarly, *MP*^*vmax*^ was the multple of v_max_, e.g. MP^vmax^ for “11M vs 6M” was 10.61 ÷ 13.15 = 0.81. MP^75^ and MP^vmax^ values were given in bold.

### Period before rapid viability loss was diagnostic

NPB-6M, NPB-11M and NPB-17M loss viability rapidly once their GP reduced to below 75% (Fig. 1a). The reduced length of t_75_ (the period for NPB-6M’s GP to drop to quarter-mortality) either due to RTS or due to priming came close to the reduced mount of t_~50_ (the period to drop to ~ half-survival level) (Table 4).

The comparison of NPB-6M to NPB-11M, to NPB-17M or to NPB-6M-S1 can be reflected not only by difference of t_75_ but also the relative multiple of t_75_. For instance, the multiple of NPB-6M’s t_75_ against NPB-11M’s t_75_ was 2.18 which meant that 5-months-longer RTS reduced t_75_ by more than half. The average rate of losing viability during t_75_, v_75_ could be estimated by dividing accumulated GP loss (reduced by ~ 20%, from ~ 95 to ~ 75%) by the period, t_75_. So, the ratio of v_75_ against others was the reciprocal of the ratio of t_75_ against the corresponding sample (Table 4). The ratio of v_75_ against a certain sample was much higher than the ratio of v_max_ (Fig. S5) against the corresponding sample (Table 4), indicating prolonged RTS affected v_75_ more than it affected v_max_. NPB-11M’s v_max_ was even lower than NPB-6M but could not compensate the effect of v_75_. The stage before significant decline of GP was predictive of seed longevity.

### Priming impaired membrane integrity at the very beginning of artificial-ageing

**Figure 4:**
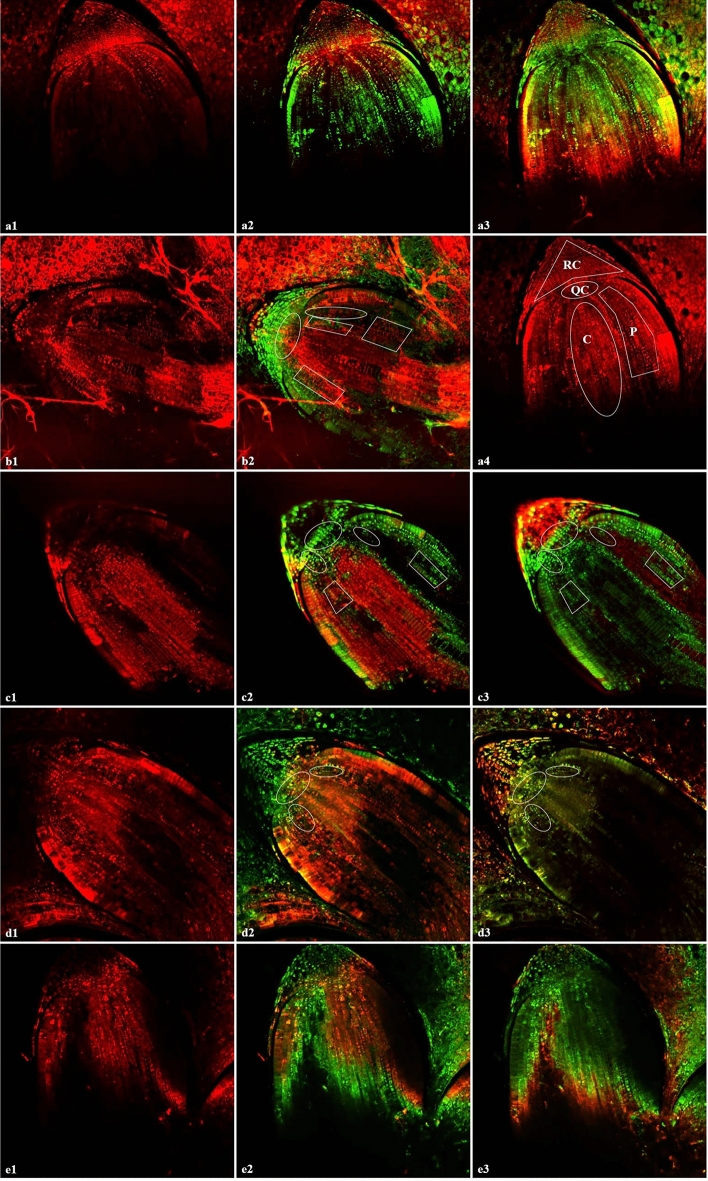
Identification of cell membrane integrity staining. In each line, the scanned layers of one sample were listed as from the surface layer (cut layer) down to the deeper layers of each exhibited embryo. Although the fluorescent staining was in red, the second and forth scanned layers were set green for comparison. By comparing red and green colors of neighbor layers, whether the deeper layer was stained positive could be seen. **(a1)**, **(b1)**, **(c1)**, **(d1)** and **(e1)** were the first layer. **(a2)**–**(e2)** were the overlaps of the first and the second (labeled green to show the comparison to the upper layer) layers; and **(a3)**–**(e3)** were the overlaps of the second and third layers, and so on. Frames labelled the positive parts where cells beneath the “seemingly positive” cut layer were still positive (not for line a and e). Positiveness of staining was assigned from 4–0 represented by line a–e respectively. **(a4)** in line b: grouping of radicle cells: *RC* root cap, *QC* quiescent center, *C* cylindrica, *P* parenchyma. But cylindrical cells were not included in scoring for 2 reasons: (1) they were much smaller and closer to each other than cells in other parts and thus easier to be stained and (2) this part was missing in some samples. Value of 4: almost the whole embryo were stained, line a, (from NPB-6M-HP); 3: most cells around the QC or joint to RC were stained and more than half of the parenchyma area was stained, but the rest of parenchyma area was still negative, line b; 2: most cells around the QC or joint to RC were stained, less than half of the parenchyma area was stained and cells within parenchyma were only occasionally stained; line c; 1: only cells near the QC or joint to RT were stained, line d; 0: hardly any cell could be stained, line e. Brightness of these images was slightly adjusted to make the layer-to-layer comparison clearer.

As AAA proceeded, rice radicles gradually became TTC-negative (unstained) (Fig. S6), which expressed the loss of local cell viability. Almost every tested NPB-11M-15d (GP = 29%) seed was labelled negative in radicle, in distinct contrast to control. Since the radicle seemed the most sensitive tissue to AAA, analysis of CMI staining was undertaken on radicles. FM4-64 is for membrane staining^24^ and vigorous cells are stained negative for the impermeability of their plasma membrane (Fig. 4). The first layer of the slices of embryo was always stained positive for mechanical damage allows the entry of the fluorescent probes. So, the extent of staining of the layer beneath the cut layer was scored instead of the surface layer.

Both H_2_O_2_- and hydropriming caused significantly higher membrane permeability than non-primed NPB-6M, hence lower membrane integrity. But the distribution of the staining levels in NPB-6M-HP was not uniform, no sample was near the average level but they usually had extreme values, 4 (Fig. 4, line a) and 0 (Fig. 4, line e). NPB-6M by contrast was mostly negative (Fig. 4, line e) or with the value of 1.

Comparing NPB-6M and NPB-6M-HP at the very beginning, it was CMI instead of ROS level that responded to post-AAA viability, for non-primed seeds had the lower impermeability than primed ones. Surprisingly, EC test (Table 5) contradicted the result of membrane-staining (Fig. 4). Although hydroprimed seeds had lower relative electrical conductivity (REC), according to Table 5, it can be calculated that this difference was due to leakage of electrolyte during priming instead of loss of CMI, for the difference between the maximum EC (from boiled samples) was likely to be attributed to priming-caused decline of EC. Including this difference in primed-samples, NPB-6M’s REC was even lower than the non-primed counterpart. Priming increased the EC through leakage during germination: the water inside the box with seeds had significantly higher EC than the water without seeds (EC-blank).

**Table 5:** Comparision of relative electrical conductivity between NPB-6M and NPB-6M-HP.

	EC-27 h	EC-boiled	REC		
NPB-6M	160.01 ± 11.68	460.86 ± 4.93	0.347	EC-priming	98.5 ± 5.2*
NPH-6M-HP	122.66 ± 8.09	418.035 ± 23.52	0.294	EC-blank	74.2 ± 1.1
Dif	37.35	42.825	0.358^#^		24.3

*EC-27 h* electrical conductivity at 27 h of soaking, *EC-boiled* electrical conductivity of fully boilled samples, ^*#*^*REC* (relative EC) of NPB-6M-HP was calculated by adding 42.825 to 122.66, *EC-priming* the EC of water in the germination box at the end of seed priming, *EC-blank* the same as EC-priming but no seed was in the box. Unit for EC: μS/cm.

*: EC-priming was significantly higher than EC-blank (P < 0.01).

## Discussion

### Antioxidant priming can prolong seed longevity before initial loss of GP

Traditionally priming is applied to pre-sowing seeds to invigorate them or trigger resistance against stresses such as drought, salinity, cold or disease^6,23,24^. Evidence of its potential to improve the longevity of high viability seeds is scarce. The significance of priming around the end of the resistant stage is that it maintains seed viability at a high level for a longer period, postponing seed viability loss.

The loss of ageing resistance is difficult to detect due to the lack of change in GP, though decline in GP is of great concern and much easier to identify. Although seeds keep high GP for decades or even more in genebanks under cold storage, the process of losing viability in this study argues for measures not only to monitor seed viability but also monitor ageing resistance. Monitoring and improving ageing resistance are more urgent for seeds preserved with poor infrastructures, which relies more on seed enhancement when long-term storage is not readily available.

To determine whether an accession is at the vulnerable stage depends on the trend of the survival curve under AAA. This can be done by comparing the curves of non-primed seeds and hydroprimed seeds to estimate whether priming prolongs or curtails seed longevity. However, determining survival curves can be time- and labor-costing.

Therefore, GP monitoring itself can be an alternative: once high viability seeds are already vulnerable to ageing, they are likely to lose viability much more rapidly later due to ROS accumulation. Notice that NPB-17M lost its ageing resistance in AAA, but is still at the plateau stage under RTS. Further, it is widely accepted that the viability loss accelerates from the beginning of initial decline of GP, reached a maximum level at GP = 50%^6^. In this way the vulnerable stage can be deduced from regular GP monitoring after initial loss of GP occurs.

### Antioxidant priming acts as a double-edge sword on seed survival

For primed seeds, redox state did affect subsequent survival: the lower the ROS accumulation, the higher viability and vigor retained under the same duration of AAA. Both HP and spd-priming which removed ROS surpassed non-primed NPB-17M whose “plateau” under AAA was not visible.

However, priming curtailed the longevity in NPB-6M along with the length of its “plateau”. What is surprising is that antioxidant priming almost equalized the viability/vigor loss rate of NPB-6M and NPB-17M, while the rates should have been quite different without priming. The effect of RTS on ageing resistance was almost eliminated, partially in accordance with *Rhododendron griersonianum* where seeds of different quality had the post-priming survival curve fitted together^25^. Prolonged seed longevity after priming was most probable in low-vigor seeds. For highly vigorous seeds in the resistant stage in this study, antioxidant priming impaired subsequent survival of rice seeds. Loss of CMI was one reason.

The resistant stage was shown to be abbreviated both by storage and priming. Non-primed, artificial-ageing resistant seeds (NPB-6M-8d) tolerated 8-days AAA and their GP still kept above 90% while their spd-primed and hydroprimed counterparts declined to GP = 75.33% and 60%, respectively, after 6-days AAA, far below NPB-6M-6d and NPB-6M-8d. This suggests that resistance to ageing is abated by priming.

Whether antioxidant priming decelerates seed deterioration for those in the vulnerable stage is complicated, depending on a specific set of factors including the solutes and their corresponding concentration. As for one popular antioxidant—ascorbic acid (AsA), its acidity can be toxic^26^. Polyamines (PAs, including spd) could act not only as ROS scavenger^27^ but also as ROS stimulator^28^. Therefore, to maximize the benefits of priming, their exact concentration of solutes should be experimentally determined. Spd at the concentration around 1 mM seemed beneficial to rice^29^. HP is much simpler for there is no solute.

### Maintenance the cell membrane integrity at the early stage of seed ageing

This study showed that the longevity for GP > 75%, t_75_ (v_75_) was a more pivotal contributor to seed longevity than the maximum viability loss rate under AAA (v_max_). So, what happened to the seed below the quarter-mortality viability contributed much less to longevity, because the curve became sharp and timespan became relatively short. The physiological state at the early stage of seed ageing seemed more essential than at the time of considerable loss of seed viability. This argues for measures to eliminate ROS and protect cell membrane in the early stages.

One explanation for the significance of CMI is that damage in the plasma membrane is followed by necrosis, a form of cell death much more dramatic and detrimental than programmed cell death^30,31^. Leakage of intracellular soluble constituents into extracellular space due to membrane permeability is one reason^32^.

Considering CMI protection, two phases can be distinguished, the pre- and post-imbibition phase. Before imbibition, the seed is dehydrated and there is hardly any metabolic or enzymatic activity. A suitable degree of dehydration induces a glassy state which stabilizes the cell membrane even in room temperature, and this can be used equally for hermetic storage under chilling or room temperature^33^.

After imbibition, the cell membrane may suffer both oxidative^20^ and mechanical damage^21,34^. The latter probably occurs during imbibition and re-drying which compose the process of priming. Although during imbibition a process of repair occurs in the cell membrane^35^, it simply means that CMI in hydrated seeds is greater than in dry seeds. It does not necessarily mean that CMI in hydrated post-priming seeds is greater than hydrated non-primed seeds.

To decrease this cost of antioxidant-priming, osmopriming (probably with polyethylene glycol which alleviates osmotic pressure on the cell membrane) is a hopeful way both for imbibition^34^ and re-drying^21^. ROS scavengers which alleviate oxidative stress through molecules like ascorbic acid, polyamines (including spd), N-Acetyl-L-cysteine can be resolved in polyethylene glycol or LiCl solution.

Further we argue that EC as an indicator for post-priming CMI is unsuitable to compare non-primed seeds directly to primed seeds, because priming itself could cause leakage of electrolytes. It creates a non-equal start for non-primed seeds which do not lose leachates during priming, though we are currently unable to explain where these leachates have gone. Maybe they were adsorbed by filter paper or the wall of the plastic box.

### Priming is complementary to seed storage and seed regeneration

Seed longevity can be increased both by storage and seed enhancement, two steps which depend on distinctly different factors. The resistant stage during which seeds are dehydrated is ideal for storage. At this stage, a pool of factors can affect seed ageing resistance: dormancy or degree of ripening^36,37^, composition of oligosaccharide or sucrose^38^ which assist the creation of a glassy stage^33^, pre-storage dehydration, and longevity genes^39^.

Benefits of priming are for rehydrated seeds, mainly from DNA repair^40^, ROS scavenging, utilization of carbohydrate^16,41,42^ and acclimation induced by stress signaling^23,24^.

Up to now, seed priming is still not widely accepted as a useful way to prolong seed longevity and its benefit is not adequately realized. In genebanks, for instance, regeneration is required probably when seed GP declines to ~ 85%^43,44^ of their original level. In between the initial loss of viability and regeneration, there are hardly any measures taken to prevent deterioration during storage. Antioxidant priming could remove ROS accumulated during storage and further prolong seed longevity, and thus postpone seed regeneration.

Priming also assists seed regeneration. It could be more crucial for precious, rare or endangered germplasm^45^ where even slight improvement can be beneficial because the population size is already small and genetic integrity can be limited. Non-random viability selection^46^ causes further genetic diversity loss and seed priming could rescue individuals as genetic sources for propagation. This increase may have a significant marginal contribution to small-size populations for regeneration. Priming can be complementary to both seed storage and seed regeneration (Table 6).

**Table 6:** Prospects of cold storage itself, antioxidant-priming compliment to cold storage and regeneration between cycles of cold storage, by comparing their pros and cons.

	Cold storage	Antioxidant priming	Regeneration
Time window	Universal	broad time window, from GP = 28% (even lower) to ~ 95%, but avoid the resistant stage	GP > 85% is probably required^43,44^; but it is universally useful because every saved seed has its value
Pros	Longevity for GP > 85 can last for decades or hundreds^4^ of years	Both longevity and viability can be improved significantly^6^; for longevity at high-viability, priming should be applied in the vulnerable stage. Priming can mitigate viability selection^46^ and genetic erosion^4^ during regeneration	It thoroughly perfects both longevity and viability; maintains genetic integrity probably when GP > 85%
Cons	Expensive, energy-costing; ultra-dry storage may be a complement or an alternative^8,33^	Injury during imbibition^34^ or desiccation^21^; loss of drought resistance^61^, leakage of abscisic acid and other resistance-related phytohormones into water	Time and labor cost, viability selection, risk of genetic pollution (interpolation with non-target gene) and genetic erosion (individual dies before reproduction)

### Survival curve, an indispensable context for timing for antioxidant priming

Evidence of benefits for post-priming survival is scarce. To our knowledge, results supporting an anti-deterioration effect of priming have probably only been reported for seed lots in the vulnerable stage^6,25,47–49^. For NPB-17, non-primed seeds were sensitive to ageing and no “plateau” took out at the survival curve. It may well be that more agreement on post-priming survival would have been reached were the survival curve of non-primed seeds taken into account. The curve can be the context to judge whether priming should be applied because it is indicative of ageing resistance: seeds with the GP beginning to decline could hardly be ageing resistant.

It is worth mention that the results that primed seeds surpass the non-primed counterpart can not necessarily lead to assume that priming is suitable to apply. Dormancy is one alternative reason why priming promotes germination^50^. Unlike previously assumed, Nipponbare in this study did have a degree of dormancy^51^ and failed to reach 50% germination immediately after harvest. Either storage or AAA^52^ could improve GP. This stage is far away from the vulnerable stage and the rise of germination after priming is not due to rise of seed viability. Some seeds are invigorated as a result of priming and a plateau stage is required to eliminate this alternative possibility.

Therefore, no priming until the detection of significant loss of resistance by determining survival curves could be a useful approach. Many survival curves have a plateau, either under AAA^6^, ambient temperature storage^5^ or cold storage^4^. Slight decrease in germination after a plateau, for instance, from 95 to 90% could mean significant loss of viability. In order not to misjudge this chance for priming and to avoid the negative effect of priming on highly ageing-resistant seeds, germination tests must be performed with considerable precision and accuracy to conclude that the decline is significant, not simply a fluctuation or an error. Now that high-throughput seed phenotyping is available^53^, seed germination can be worked out with cameras and computers as precisely as manual work^54^, and it is much easier to determine survival curves for a large number of seed accessions. For seeds stored under room or ambient temperatures which deteriorate much faster than cold storage, there can be greater need for seed enhancement. Further, the timespan to determine a survival curve under normal storage is much less than for cold storage. So, the practice to improve seed longevity by priming can first be applied to short-term storage in ambient temperatures.

Despite the great prudence concerning the effect of priming on high-viability seeds, a broader time window for low-viability seeds is worth determining because it probably not only decelerates deterioration but also increases seed viability^6^. Seed longevity is the timespan for its GP to decrease to a given level, e.g., 75%, t_75_. An increase in GP alone, for instance, from 75 to 80%, regardless of the post-priming deterioration rate, at least increases the t_75_ from zero.

Since priming has more advantages for low-quality seeds than higher ones^6^, the time window for priming seeds who lose viability can be as broad as seed GP dropped to ~ 30% according this study. For a wide range of GP for priming, it is likely to combine several cycles of seed enhancement, and the possibilities are infinite. For instance, 3 cycles of osmopriming increased t_85_ (the timespan for seeds to reduce to GP = 85%) to more than double in *Digitalis purpurea*, while 2 cycles respectively at GP≈85% and GP≈80% doubled t_75_^6^.

Regarding our study in rice, repeated priming seemed harmful. Perfecting the re-drying and combining antioxidant chemicals in osmopriming to avoid cell membrane damage may be a measure to realize the benefits of repeated priming. Shortening the imbibition duration may be another prospective choice for seeds of high vigor^6^. The reason lies in that the best time to end imbibition was supposed not far before radicle protrusion to maximize the benefits of physiological processes, e.g., DNA repair^6^ and ROS remove^55^. Indeed, before protrusion a series of germination process advances including the entry of a mass of cells into the S-stage, and tiny embryonic elongation which is too hard to identify^56^. Seeds gradually lose desiccation tolerance as germination advances. High vigor seeds’ imbibition may reach a stage where their desiccation tolerance vanishes but low vigor seeds did not reach that stage^6^. Change of hormonal metabolism during imbibition is another possible reason which is very complicated^57^. The possibilities of priming’s negative effect are unlimited and that may be the limitation for its application. In practice maximizing its benefits has to be based upon the indispensable context: the survival curve.

Cold plasma is a recently well-advanced seed enhancement without the process of imbibition. Its combination with priming is also worth study for it smoothens seed surface^58^, adjusts osmotic solutes and changes the subsequent process of water absorbation^59^, induces antioxidant response, and also disinfects seeds^59,60^.

This study is, to our knowledge, the first article distinguishing the double effect of priming on seed lots of almost the same viability and vigor but of distinct ageing resistance. However, this article only focused on the antioxidant effect of priming at high GP level and only one crop variety was tested. Timing and planning for antioxidant priming have the potential to extend priming to other cultivars, species and circumstances to assist seed preservation. The details include solutes for priming, duration of priming, post-imbibition desiccation and combinations of cycles of seed enhancement.

## Materials and methods

### Rice seed sample

Seeds of *Oryza sativa* L. subsp.geng cv. Nipponbare (NPB) were harvested in 2014 (NPB-6M and NPB-17M) and 2016 (NPB-11M)^61^ in Nanchang, Prov. Jiangxi. Four mo. after harvest their GP was ~ 50%. NPB-6M, 17M and 11M were under room-temperature-storage (RTS) in a resting room of the National Genebank in Beijing for 6, 17 and 11 mo. respectively. Before RTS they were kept at − 18 °C and then kept at 4 °C after RTS until the test. The room temperature ranges from ~ 20 to ~ 30 °C with the help of a heating system which works for 4 mo. in winter along with air conditioners.

Seeds were air-dried to 10.71% moisture content (w(H_2_O)/w(DW), gravimetrically determined by comparing the weight of ground seed powders before and after heating at 105 °C for 6 h^62^). Seeds underwent AAA at 40 °C and 75% relative humidity (RH, water vapor of saturated NaCl solution) for diverse durations, e.g. 6 days for NPB-6M-6d and NPB-17M-6d, and 10 days for NPB-6M-10d and NPB-17M-10d; and so on for – 4 days, − 8 days, − 12 days, − 14 days, − 15 days, − 17 days, − 19 days and – 20 days (− 1 days, − 3 days, − 5 days, 5.5 days specially for NPB-11M). Seeds were sealed in aluminum foil bags under ~ 5 °C before use. Further treatment information is in Table 1.

### Germination tests and priming treatments

GP was examined in a 7-day germination (28 °C, dark, wet; 50 seeds per box and more than 2 boxes per sample regarding seed scarceness). Following ISTA^63^, a seed 7 days after sowing that was guaranteed to be a seedling was counted as germination when abnormal germination was excluded from the final GP. Priming was done in the same way as germination in the first 24 h which is supposed to be ahead of the protrusion of an embryo. After the 24 h the seeds were collected, rinsed with distilled water, dried on paper towel and dehydrated on silica gel (~ 11% RH, 48 h) to ~ 8.5% which is significantly higher than 5%, a widely accepted safe level (the real water content was a little higher than 8.5% because during weighting very little biomass can be lost during operation, e.g., friction of the coat). Post-priming water content was worked out with the original water content, (10.71%), the weight of a seed lot (a little more than 100 seeds) measured before and after priming. For certain treatments seeds were incubated in spd solution as antioxidant priming or in H_2_O_2_ solution as oxidant priming instead of pure water. VI = W × GI, VI was the product of GI and average per-capital dry weight (mg) of shoot and root, W. GI = ∑(Gt/t) × 100 ÷ 50, Gt is the day-by-day germination percentage at day t, and 50 means that each replicate contains 50 seeds.

### Histochemical staining

Seed embryos were excised, longitudinally dissected with a blade and then incubated in triphenyltetrazolium chloride (TTC, 2%) and nitro blue tetrazolium chloride (NBT, 5%) for 30 min (37 °C) for cell viability test, ROS measurement respectively and photograph was taken either by a camera or by a scanner. The similar process was for excised embryos incubated on ice for 10 min (endocytosis is supposed to occur after 10 min^64^) with the fluorescent probe FM4-64^35,65^ (Invitrogen, 5 mg/L) to measure CMI.

### Raking of CMI according to microscopic images

Labelling was identified for CMI by photographing with a confocal laser scanning microscope (Leica SP8, excitation wavelength: 552 nm, emission wavelength: 599–651 nm; the intensity of the light was similar in all the samples to avoid bias). Scanning was from the surface layers to deeper layers to check whether the layer beneath the stained wound layer was also stained. Almost every tested GP = 30% seed had a dead radicle according to TTC staining, in distinct contrast to control (Fig. S6). Since radicles seemed the most sensitive tissue to AAA, analysis of CMI staining was undertaken on radicles by analysis in four tissues: root cap, quiescent center, cylindrica and parenchyma (Fig. 4). Vigorous cells are stained negative for the impermeability of their plasma membrane (Fig. 4). The first layer of the slices was always stained positive for mechanical damage allows the entry of the fluorescent probes, so the extent of staining of the layer beneath the cut layer was scored instead of the surface layer (Fig. 4, Video S1). Post-AAA samples, NPB-6M-S1/HP/H100-6d were not analyzed because it is very complicated to judge whether the damage was the consequence responding to the initial state during ageing or was it one of the causes leading to deterioration.

### Electrical conductivity test

Twenty five seeds as a replicate were soaked in 5 ml distilled water in a 10 ml microtube and kept in 28 °C (germination temperature) and EC was measured (Delta326, Mettler-Toledo, China) at 0 h without seeds as a blank. The tubes were then filled with 25 seeds each and EC was tested in each tube chronologically at 18 h, 21 h, 24 h and 27 h to select a stable state to calculate final EC and ultimately after EC became stable, by putting tubes in a boiling-water bath for 25 min after EC, REC could be drawn. NPB-6M-HP contained four replicates and non-primed sample, NPB-6M contained two replicates because of the lack of enough cohort seeds (seeds shared exactly the same experience) and because the variation between non-primed replicates was supposed to be less than primed ones (priming itself could cause variation in leakage of electrolyte). During the process of priming, hydroprimed seeds were also tested for EC but they were placed on filter papers in a plastic germination box as being primed. The same kind of box with 10 ml water and the two filter papers was also tested for EC at 24 h as a counterpart to boxes containing seeds to check whether priming caused greater EC of water in the box.

### Data analysis

Analysis of variance (ANOVA) was performed with SPSS and values were expressed as MEAN ± SE (standard error). Significance of difference was checked by LSD-test for treatment data which met homogeneity of variance (p > 0.05 for Levene statistics). Other data were transformed, and no data finally failed to meet homogeneity of variance for LSD-test after transformation either by log-transformation or by square-root-transformation. Percentages were arcsin-transformed only for multiple comparison test. The maximum rate of seeds to lose viability was modelled by curve fitting with Graphpad Prism (Graphpad Software Ins, La Jolla) using a logistic regression^66^. This model supposes a normal distribution of survival along the time axis of ageing and that the rate of losing GP per day, accelerates from the very beginning of initial loss of viability and peaked out at GP = 50%, hence the v_max_. Logistic regression is quite similar to and more explainable than the popular probit regression^6^ whose y-axis is not simply the even distribution of germination percentage. Therefore, logistic model makes viability loss rate more apparent than probit model. Viability loss rate at viability level other than GP = 50% was estimated by dividing accumulated loss of GP by the duration of AAA (% day^−1^). Longevity to drop to GP = 75% or 40–60% was expressed as t_75_ and t_~50_ respectively.

## Supplementary information

Supplementary Video.

Supplementary Information.
