# Supplementary material for: Timing for antioxidant-priming against rice seed ageing: optimal only in non-resistant stage
Source: Sci Rep. 2020 Aug 6;10:13294. doi: 10.1038/s41598-020-70189-6 (PMC7411016; doi:10.1038/s41598-020-70189-6)
Supplement: Supplementary file 2 — Supplementary Information. [file 41598_2020_70189_MOESM2_ESM.docx]

Timing for antioxidant-priming against rice seed ageing— optimal only in non-resistant stage

Ling-xiang Xu^1,2,3^, Xia Xin^1^*, Guang-kun Yin^1^, Ji Zhou^3^, Yuan-chang Zhou^2^, Xin-xiong Lu^1^*

1. National Crop Genebank, Institute of Crop Science, Chinese Academy of Agricultural Sciences, Beijing 100081, China

2. Key Laboratory of Ministry of Education for Genetics, Breeding and Multiple Utilization of Crops, College of Crop Science, Fujian Agriculture and Forestry University, Jinshan, Fuzhou 350002, Peoples R China

3. Plant Phenomics Research Center, Nanjing Agricultural University, Nanjing 210095, Peoples R China

Table S1 Primary selection of solutes for seed priming for NPB-17M using germinability and vigor after post-priming ageing.

|  |  | AAA 0 d | | |  | AAA 10 d | | |
| --- | --- | --- | --- | --- | --- | --- | --- | --- |
|  | N GP±SE (%) | | N VI±SE n.s. | | N GP±SE | | N VI±SE | |
| H100 | 4 | 92.96±1.92 bcd(bc) | 4 | 107.69±7.87 | 3 | 13.33±4.37 b(b) | 3 | 7.65±2.57 b(b) |
| H50 | 2 | 94.50±1.71 bc(abc) | 2 | 104.85±9.95 | 3 | 14.00±2.67 b(b) | 3 | 6.34±4.81 b(b) |
| S5 | 4 | 92.50±0.96 cd(bc) | 4 | 96.93±2.19 | 3 | 40.00±2.00 a(a) | 3 | 28.19±0.62 a(a) |
| S3 | 4 | 97.50±0.50 ab(ab) | 4 | 93.86±7.09 | 3 | 44.00±3.06 a(a) | 3 | 27.04±9.11 a(a) |
| S2 | 4 | 96.52±0.49 abc(ab) | 4 | 104.84±6.26 | 3 | 38.00±4.00 a(a) | 3 | 25.37±0.97 a(a) |
| S1 | 3 | 96.69±1.29 abc(ab) | 3 | 110.34±2.90 | 3 | 38.00±6.11 a(a) | 3 | 27.79±3.54 a(a) |
| S0.5 | 4 | 95.00±2.38 f.h. | 4 | 100.76±10.78 | 3 | 43.33±1.33 a(a) | 3 | 22.90±0.33 a(a) |
| S0.25 | 4 | 89.03±2.11 d(c) | 4 | 103.44±6.41 |  | n. |  | n. |
| HP | 4 | 96.00±2.00 abc(abc) | 4 | 98.22±3.61 | 2 | 36.00±2.31 a(a) | 2 | 24.05±2.25 a(a) |
| NP | 5 | 98.00±0.63 a(a) | 2 | 105.58±3.91 | 5 | 11.33±4.37 b(b) | 2 | n. |

f.h., data which caused failure of homogeneity test in variance analysis was excluded from multiple comparison. NP, no priming. HP, hydropriming. GP, germination percentage. SE, standard error. VI, vigor index. H100, 50, priming with 100, 50 mM hydrogen peroxide. S5–S0.25, priming with 5 mM –0.25 mM spermidine. Different letters indicated that the levels were significantly different with P<0.05 (extremely significantly with P<0.01 for letters within the brackets). *,**:significantly higher than non-primed control at P＜0.05，0.01 level respectively. #:significantly lower than control at P＜0.05.

Table S2. Effect of H_2_O_2_ concentration.

|  | N | GP±SE (%) | N | GP.Ab±SE(%) |
| --- | --- | --- | --- | --- |
| NPB-17M-H50-10d | 3 | 13.33±4.37 | 3 | 20.67±6.77 |
| NPB-17M-H100-10d | 3 | 12.67±2.67 | 3 | 15.33±2.91 |

GP: germination percentage. GP.Ab: percentage of abnormal germination plus normal germination. SE: standard error. Although no significant difference existed between these treatments, 100 mM H_2_O_2_ caused lower GP.Ab and was selected for its potential negative effect on seed viability.

Table S3. Effect of spermidine-priming and hydro-priming on seeds whose GP was over 80%.

|  | N | GP±SE(%) |  | VI | N |  | GP±SE(%) |  | VI |
| --- | --- | --- | --- | --- | --- | --- | --- | --- | --- |
| **17M** | **5** | **98.00±0.63** |  | **105.58±3.91** | **4** | **6M-10d** | **82.33±0.81** | **2** | **74.90±0.01** |
| 17M-S1 | 3 | 96.69±1.29 |  | 110.34±3.35 | 4 | 6M-10d-S1 | 84.00±0.00 | 4 | 80.92±0.42 |
| 17M-HP | 2 | 96.00±2.00 |  | 98.22±3.61 | 2 | 6M-10d-HP | 89.00±1.00 |  | n. |
| **NPB-6M** | **4** | **96.69±1.29** |  | **105.28±2.38** |  |  |  |  |  |

GP: germination percentage. VI, vigor index. SE: standard error. n., no data. HP, hydropriming. S1, priming with 1 mM spermidine. NPB-6M’s viability and vigor were between NPB-17M and NPB-6M-10d. Both spd-priming and hydropriming showed no significant effect on seed survival. Supposing priming could only have negative effect for seeds of viability or vigor higher than NPB-17M, it would not have negative effect on NPB-6M. Therefore, at 0 d, NPB-6M and NPB-17M probably had the same viability and vigor if they were primed in spermidine or pure water.





Figure S1. Curve fitting to compare the initial day of loss of germinability of NPB-6M by comparing it to NPB-17M and -11M.

In both a and b one single curve was drawn to fit all the points. The supposed day of initial germinability loss caused the best fitting at 8 d and 6 d in a and b respectively when the R value was the maximum.





Figure S2. Deterioration curve of NPB-17M and NPB-17M-HP.

GP, germination percentage. VI, vigor index. Dot lines indicated GP=75% (start of rapid deterioration) and GP=40% (~ GP of NPB-17M-S0.5/S1/S2/S3/S5/HP-10d in Tab. 2). The GP of the tested sample with the ideal duration of artificial ageing should be between 75% and 40% to make comparison to other treatments and 6d seemed optimal. Standard error was labeled by bar.





Figure S3. Deduction of the range of germination percentage and vigor index of NPB-6M-S1/HP/H100-6d.

GP, germination percentage. VI, vigor index. GP of NPB-6M-S1-10d was close to that of NPB-17M-S1-10d, so was VI. So, supposing this trend was the same with NPB-6M-S1-6d and supposing NPB-6M and NPB-17M seeds under the same priming treatment and the same duration of ageing had the similar GP and VI, the range of GP and VI of NPB-6M-S1/HP/H100-6d could be deduced and were labeled by interrogation with green for S1, blue for HP and orange for H100. Standard error was labeled by bar.


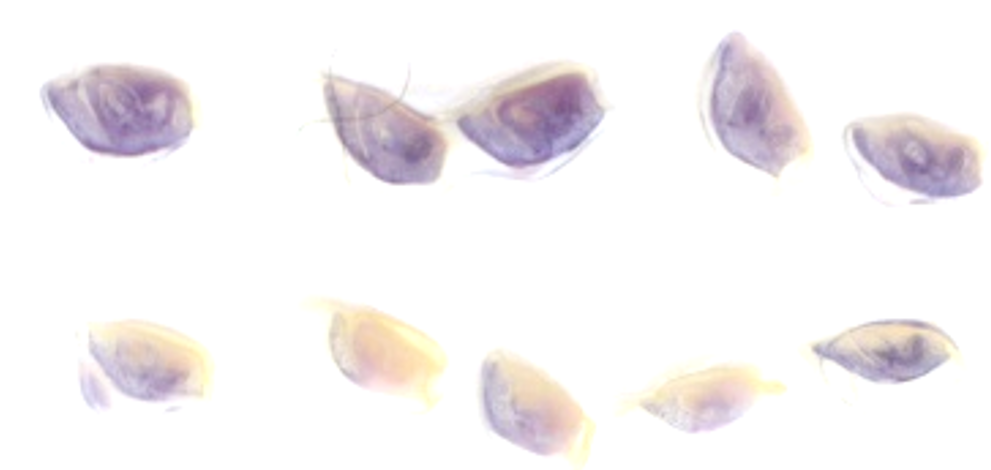


Figure S4. ROS labelling of deteriorated seeds (GP=29%) with and without post-ageing hydropriming.

Seed embryos were dissected, and incubated with nitro blue tetrazolium chlorid (5%, 20 min, 37 °C). The upper line of seeds experienced post-ageing priming and the lower line not.





Figure S5. Curve fitting to deduce maximum deterioration rate at the half-survival level.

GP, germination percentage. Diamond shaped dots of NPB-11M were from another accession harvested in 2014 under room-temperature storage for 17 mo. to improve curve fitting. Modeled maximum deterioration rate (v_max_) for NPB-6M, 11M, 17M, and 17M-HP was 13.15, 10.61, 24.76 and 17.39 % d^-1^ respectively and the regression value of fitting was 0.9934, 0.9788, 0. 9994 and 0. 9848 respectively.


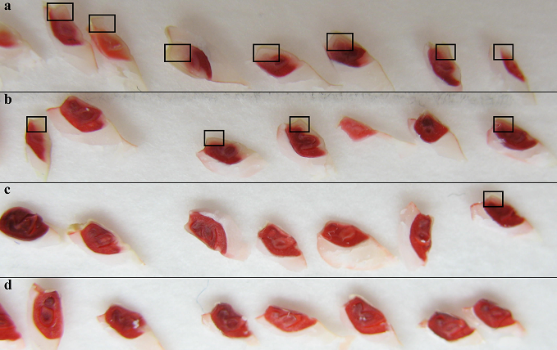


Figure S6. TTC staining in radicles of NPB.

Frames indicated radicles that failed to be stained by TTC. a, GP=36%; b, GP=53%; c, G=84%; d, GP=95%(control). Seed accession was the same as NPB-6M which was harvested in 2014, but these seeds experienced 11-mo. room-temperature storage
